# Forensic-oriented injury and abnormality assessment in sports medicine via a biomechanically-informed predictive framework

**DOI:** 10.3389/fmed.2026.1759763

**Published:** 2026-05-01

**Authors:** Xiaolin Wang, Liduan Zheng, Zeyu Li

**Affiliations:** 1 Department of Pathology, Union Hospital, Tongji Medical College, Huazhong University of Science and Technology, Wuhan, China; 2 School of Life Sciences, Northeast Forestry University, Harbin, China

**Keywords:** biomechanical optimization, injury risk prediction, machine learning, multimodal data integration, sports medicine

## Abstract

**Introduction:**

Injury assessment and forensic decision support are pivotal challenges in sports medicine, requiring advanced methods to interpret complex biomechanical and medical imaging evidence under uncertainty. This study presents the Biomechanical Informed Predictive Optimization Network (BIPON), a machine learning framework designed to support evidence based injury and abnormality assessment, with a general structure that accommodates multimodal data sources, including visual, temporal, and auxiliary information.

**Methods:**

The framework comprises three conceptual components: the Biomechanical Data Integration Module (BDIM), the Injury Risk Prediction Module (IRPM), and the Performance Optimization Module (POM). In this manuscript, BIPON is instantiated and empirically evaluated in an imaging based setting, focusing on exam level injury and abnormality assessment using public knee MRI benchmarks. The proposed model employs hierarchical feature fusion and adaptive biomechanical feature weighting to improve discrimination, calibration, and robustness of imaging based predictions, which are critical for forensic documentation and clinical decision support. While BIPON is formulated to support multimodal injury risk modeling and biomechanically constrained performance optimization, these components are included as formally specified extensions of the framework and are not claimed as empirically validated in the present study due to data availability constraints.

**Results and discussion:**

Experimental results demonstrate the effectiveness of the proposed approach on benchmark based imaging assessment tasks, and the optimization module is described as a reproducible constrained formulation intended for future validation when datasets with controllable action variables and measurable performance outcomes become available. In a forensic context, injury risk assessment primarily concerns evidence based evaluation of injury presence, severity, and uncertainty at the time of examination, rather than prospective outcome forecasting.

## 1 Introduction

Sports medicine has increasingly embraced technological advancements to address injury risk prediction and performance optimization, driven by the growing demand for personalized healthcare solutions. The integration of biomechanical and forensic data offers a unique opportunity to enhance the accuracy and reliability of predictive models, not only enabling early detection of potential injuries but also optimizing athletic performance through tailored interventions (1). This task is essential as traditional approaches often fail to account for the complex interplay between biomechanical factors and forensic data, limiting their effectiveness in real world applications. Moreover, the rapid evolution of machine learning techniques provides a promising avenue to overcome these limitations, offering scalable solutions that can adapt to diverse scenarios (2). By leveraging these advancements, researchers can not only improve the precision of injury risk assessments but also contribute to the broader field of sports medicine by fostering innovation in performance optimization strategies. From a forensic medicine perspective, injury risk assessment focuses on the objective interpretation of available evidence such as medical imaging and clinical records to support injury determination, documentation, and medico legal decision making, rather than predicting future injury incidence.

Initial efforts to predict injury risks and optimize performance relied on manually defined frameworks that utilized biomechanical principles and expert knowledge. These methods aimed to model the relationships between physical parameters, such as joint angles, muscle forces, and external loads, to identify potential injury mechanisms. While these approaches provided valuable insights into specific risk factors, they were constrained by their inability to process large scale and complex datasets (3). Furthermore, their reliance on static models limited their adaptability to dynamic and multifaceted scenarios, which are common in sports medicine (4). These challenges highlighted the need for more flexible and scalable methodologies capable of handling diverse data sources and capturing intricate biomechanical patterns.

The introduction of algorithmic approaches capable of learning from structured datasets marked a significant advancement in injury risk prediction and performance optimization. By analyzing historical data, these methods identified patterns and correlations that were previously difficult to discern. Techniques such as decision trees and ensemble methods demonstrated improved predictive accuracy by integrating data from motion capture systems, force plates, and other biomechanical tools. These models also facilitated the inclusion of forensic data, enhancing the robustness of injury assessments. However, their reliance on predefined features posed limitations, as crafting relevant features required substantial domain expertise and often failed to capture the complexity of high dimensional data, such as video recordings or sensor streams (5). This limitation underscored the need for approaches capable of directly processing raw and unstructured data.

Recent advancements in computational models have enabled the automatic extraction of meaningful patterns from complex datasets, revolutionizing injury risk prediction and performance optimization. Neural network architectures, including convolutional and recurrent networks, have shown remarkable success in analyzing high dimensional data such as motion sequences, sensor signals, and video recordings (6). Pre-trained models leveraging transfer learning have further enhanced predictive capabilities by utilizing knowledge from large scale datasets to improve performance in sports medicine applications. These approaches also support the integration of multimodal data, combining biomechanical and forensic information to provide comprehensive insights into injury risks and performance factors (7). Despite their transformative potential, these models face challenges such as high computational demands and limited interpretability, which can hinder their practical application in clinical and athletic settings (8). Addressing these challenges requires innovative strategies that balance computational efficiency, predictive accuracy, and transparency.

Based on the aforementioned limitations, we propose a method that integrates biomechanical and forensic data using a hybrid framework that combines interpretable machine learning techniques with deep learning models. Our approach aims to address the scalability and adaptability issues of symbolic AI, the feature engineering challenges of traditional machine learning, and the computational demands and interpretability concerns of deep learning. By leveraging advanced data integration techniques, our method ensures the seamless fusion of multimodal data, enabling more accurate and comprehensive injury risk predictions. Furthermore, we incorporate domain specific knowledge into the model design to enhance interpretability and facilitate practical applications in sports medicine. This hybrid framework not only improves predictive accuracy but also optimizes performance by identifying actionable insights that can guide personalized interventions. Through extensive experimentation and validation, we demonstrate the effectiveness of our approach in addressing the limitations of existing methods and advancing the field of sports medicine. This study presents a framework focused on exam-level injury and abnormality detection based on knee MRI scans from the MRNet dataset. While the framework has the potential for multimodal integration, performance optimization, and real-time deployment, these aspects are not evaluated in this study due to the limitations of available datasets.

The contributions of this work are as follows:

- A method for exam-level knee MRI assessment, focused on abnormality detection and tear identification, evaluated using MRNet and MURA benchmarks.
- A robust evaluation of imaging-based abnormality prediction with detailed statistical reporting.

## 2 Related work

### 2.1 Biomechanical data in sports medicine

The integration of biomechanical data into sports medicine has been extensively studied, focusing on its role in understanding injury mechanisms and enhancing athletic performance. Quantitative measurements such as joint kinematics, muscle activation patterns, and ground reaction forces are commonly analyzed to identify biomechanical inefficiencies that may predispose athletes to injuries (6). Advanced technologies, including motion capture systems and electromyography, are frequently employed to collect these datasets, enabling researchers to examine movement patterns in detail (9). Studies have highlighted the importance of abnormal joint kinematics, such as excessive knee valgus, in increasing the risk of anterior cruciate ligament injuries (1). Machine learning models, particularly those utilizing supervised learning techniques, have been developed to classify movement patterns as safe or risky, providing a predictive framework for injury prevention (7). Unsupervised learning approaches, such as clustering algorithms, have also been applied to uncover latent biomechanical features that traditional methods may overlook (10). Biomechanical data has further been utilized to optimize athletic techniques, with gait analysis being a prominent example in improving running economy among endurance athletes (8). Real time data processing facilitated by machine learning algorithms has enabled actionable feedback for athletes and coaches, revolutionizing training methodologies (11). Personalized interventions, accounting for individual variability in biomechanics, have been shown to enhance both injury prevention and performance optimization, underscoring the transformative potential of integrating biomechanical data with machine learning (12).

### 2.2 Forensic data for injury analysis

Forensic data provides a complementary perspective to biomechanical measurements in sports medicine, offering insights into the environmental and situational factors contributing to injuries. Retrospective analyses of injury events, often conducted through video footage and environmental monitoring, have been instrumental in reconstructing the sequence of events leading to injuries (13). Frame by frame examination of athlete movements and their interactions with playing surfaces or equipment has revealed critical insights into injury mechanisms (14). Machine learning techniques, particularly convolutional neural networks, have been employed to automate the extraction of spatiotemporal features from video data, enhancing the precision of injury analysis (15). Environmental conditions, such as temperature and surface characteristics, have been identified as significant contributors to injury risk, with studies showing higher injury rates on artificial turf compared to natural grass (16). Large scale forensic datasets analyzed through machine learning models have uncovered correlations between environmental factors and injury outcomes, informing the development of safer playing conditions (17). The integration of forensic data with biomechanical measurements has enabled a holistic approach to injury analysis, particularly for complex injuries like concussions (18). Combining video analysis with motion capture data has provided a detailed understanding of how biomechanical factors interact with external conditions to cause injuries (19). This integrated approach has proven valuable in developing targeted injury prevention strategies, ultimately improving athlete safety and performance (20).

### 2.3 Machine learning in sports medicine

Machine learning has emerged as a pivotal tool in sports medicine, offering advanced capabilities for analyzing complex datasets and generating actionable insights. Predictive algorithms trained on historical data, including biomechanical and forensic variables, have been developed to identify athletes at high risk of injury (21). Recurrent neural networks and long short term memory networks have been particularly effective in analyzing time series data, such as joint kinematics, to predict injury likelihood (22). Contextual factors, including training load and environmental conditions, have been incorporated into these models to enhance predictive accuracy (23). Beyond injury prediction, machine learning has been applied to optimize athletic performance by identifying inefficiencies in movement patterns and providing personalized recommendations (24). Reinforcement learning techniques have been utilized to simulate and refine athletic movements, generating strategies that maximize performance while minimizing injury risk (25). Wearable sensors and mobile health technologies have facilitated the continuous monitoring of athletes during rehabilitation, with machine learning algorithms analyzing data streams to track recovery progress and predict re-injury risks (9). Personalized rehabilitation programs, informed by machine learning insights, have demonstrated improved outcomes by adapting to the unique needs of each athlete (1). The integration of machine learning into sports medicine has not only advanced injury prevention and performance optimization but also enhanced the effectiveness of rehabilitation protocols, marking a significant leap forward in the field (7).

### 2.4 MRI based injury/abnormality assessment

In addition to biomechanics and wearable based injury risk modeling, a complementary research line focuses on medical imaging for injury/abnormality assessment, where MRI is widely used to identify structural patterns associated with knee injuries. Prior work on knee MRI classification has explored exam level prediction from multi slice studies using slice wise encoders with study level aggregation as well as volumetric modeling variants. Because our experimental validation is conducted on the MRNet knee MRI benchmark (exam level binary classification), we position our comparisons primarily against established medical imaging classification backbones and study level aggregation paradigms. We note that biomechanics and wearable driven injury forecasting is an important and active direction in sports medicine; however, such multimodal longitudinal signals are not available in the MRNet benchmark used for our current evaluation. Therefore, related work in wearables/biomechanics is discussed as background and motivation, while the empirical validation in this manuscript focuses on MRI exam level abnormality prediction.

We note that, while sports injury prediction using wearable sensors and biomechanical time series modeling is an important research direction, the empirical focus of this manuscript is restricted to imaging based, exam level injury and abnormality assessment. Accordingly, the related work and experimental baselines emphasize medical imaging classification, study level aggregation, and probabilistic calibration, rather than prospective injury forecasting from sensor data.

## 3 Method

### 3.1 Overview

The proposed methodology seeks to tackle the complexities of injury risk prediction and performance optimization within sports medicine by employing machine learning techniques and integrating multimodal biomechanical and forensic data. This section delineates the methodological framework, which is organized into three principal components: the Biomechanical Data Integration Module (BDIM), the Injury Risk Prediction Module (IRPM), and the Performance Optimization Module (POM). Each module is meticulously crafted to process and analyze diverse data sources, facilitating precise predictions and actionable insights pertinent to sports medicine applications.

In Section 3.2, the problem of injury risk prediction and performance optimization is formalized within the sports medicine context. This involves the definition of multimodal data sources, encompassing visual data (*X*_*v*_), temporal data (*X*_*t*_), and auxiliary data (*X*_*a*_), along with their respective feature representations (*F*_*v*_, *F*_*t*_, *F*_*a*_). The section further introduces the concept of joint feature representation (*F*_joint_), which underpins subsequent predictive and optimization tasks. By establishing a mathematical framework, clarity is provided on the integration and utilization of these data sources within the proposed pipeline.

Subsequent to the preliminaries, Section 3.3 presents the Biomechanical Informed Predictive Optimization Network (BIPON), the central model of our methodology. BIPON is engineered to process multimodal data through hierarchical feature extraction and fusion techniques, culminating in a unified representation (*F*_joint_) that encapsulates the intricate relationships among biomechanical, temporal, and auxiliary data. The model architecture incorporates specialized mechanisms to address the complexities inherent in sports medicine data, such as adaptive feature weighting and hidden state representations (*H*). This section expounds on the design principles and computational strategies underpinning BIPON, emphasizing its capacity to confront the unique challenges of injury risk prediction and performance optimization.

Section 3.7 elaborates on the innovative strategies employed to augment the efficacy of BIPON in practical applications. The initial strategy centers on adaptive biomechanical feature weighting, which dynamically modulates the significance of various data modalities based on their pertinence to injury risk prediction. This ensures the model's robustness and interpretability across diverse scenarios. The subsequent strategy involves iterative optimization of performance metrics, utilizing biomechanical constraints to refine predictions and recommendations. By embedding domain specific knowledge into the optimization process, this approach bridges the divide between theoretical modeling and practical implementation in sports medicine.

The present study addresses a cross sectional prediction setting: given a single knee MRI examination acquired at one time point, the model outputs calibrated probabilities for three study level targets defined by MRNet, i.e., abnormality, ACL tear, and meniscus tear. No prospective forecasting horizon is modeled in our experiments; the reported performance should be interpreted as cross sectional diagnostic screening/forensic triage accuracy rather than future injury incidence prediction. Therefore, no prospective forecasting horizon is modeled in our experiments, and the reported performance should be interpreted as diagnostic screening/forensic triage accuracy based on imaging evidence rather than future injury incidence prediction. We believe this setting remains clinically relevant because exam level abnormality screening is a standard entry point for subsequent clinical decision making, referral prioritization, and medico legal documentation workflows. This manuscript contains two conceptually different outputs. (i) The injury related output is an assessment/prediction score for injury/abnormality labels (in our benchmark evaluation: abnormality, ACL tear, and meniscus tear on MRNet; abnormality on MURA), which is evaluated by standard classification and calibration metrics. (ii) The performance optimization output is an optimized action/plan that aims to improve a task specific performance metric subject to biomechanical feasibility and safety constraints. Because MRNet/MURA do not provide controllable action variables or measurable downstream performance outcomes, the empirical evaluation in this manuscript focuses on the injury/abnormality assessment task and external validation; end to end validation of constrained performance improvement is left for future work on datasets with synchronized biomechanics and performance measurements.

### 3.2 Preliminaries

To avoid ambiguity between the benchmarked model and the broader framework, we distinguish throughout this section between (i) the evaluated pipeline used in the MRNet/MURA experiments and (ii) conceptual extensions of BIPON intended for future multimodal instantiations. In the present study, the empirically evaluated pipeline is a visual-only MRI-based instantiation that takes a three-view knee MRI examination as input, performs slice encoding, within-view aggregation, and cross-view fusion, and outputs exam-level probabilities for abnormality, ACL tear, and meniscus tear. Components involving temporal/biomechanical streams, auxiliary/forensic records, cross-stream synchronization/alignment, and the Performance Optimization Module (POM) are included only as conceptual framework extensions and are not used in the MRNet/MURA experiments. This subsection formalizes the problem of injury risk prediction and performance optimization in sports medicine by integrating biomechanical and forensic data. The objective is to construct a machine learning framework that utilizes multimodal data sources to predict injury risks and optimize performance metrics. The problem is defined mathematically, and the key variables and representations used throughout the methodology are introduced.

Although the full BIPON framework can be written with several auxiliary or task-specific objective terms for future multimodal extensions, the only optimization objective used in the reported MRNet/MURA benchmark experiments is per-label binary cross-entropy (BCE) applied to the three exam-level outputs. Any loss formulations below involving focal reweighting, cross-modal alignment, biomechanical constraints, or optimization-related penalties should be interpreted as framework-level options for future instantiations, rather than as losses used in the present benchmark evaluation.

Although the general BIPON framework is formulated using three generic modalities, namely visual data *X*_*v*_, temporal/biomechanical data *X*_*t*_, and auxiliary/forensic data *X*_*a*_, the empirical benchmark evaluation reported in this manuscript instantiates only the visual branch. In the MRNet/MURA setting, *X*_*v*_ corresponds to a single knee MRI examination composed of sagittal, coronal, and axial views. The public benchmarks used here do not provide synchronized biomechanical time-series signals or auxiliary forensic/clinical records at the session level; therefore, *X*_*t*_ and *X*_*a*_ are not instantiated in the reported experiments. Accordingly, any multimodal notation introduced below should be interpreted as a general formulation of the framework rather than as a statement that all modalities are used in the present benchmark experiments.

Let *X*_*v*_, *X*_*t*_, and *X*_*a*_ denote the three primary data modalities considered in this study. Specifically, *X*_*v*_ represents visual data, including video based biomechanical features captured during sports activities. *X*_*t*_ corresponds to temporal data, such as time series motion data obtained from sensors or tracking devices. *X*_*a*_ refers to auxiliary data, including forensic and medical records that provide contextual information about the athlete's condition and history. These modalities form the basis of the proposed framework. Although the BIPON framework is formulated for three generic modalities (*X*_*v*_, *X*_*t*_, *X*_*a*_) to support future multimodal sports medicine deployments, the experimental validation in this manuscript instantiates only the visual modality using the MRNet knee MRI examinations. In particular, *X*_*v*_ corresponds to a single MRI exam (a multi-slice volume) and the supervision label is the MRNet exam-level abnormality indicator. The MRNet benchmark does not provide synchronized kinematic time series or auxiliary forensic/EHR fields at the subject session level; therefore, *X*_*t*_ and *X*_*a*_ are not used in the current benchmark evaluation. We explicitly state this to avoid ambiguity and to ensure full reproducibility of the reported results. In the benchmarked visual-only instantiation used throughout this manuscript, the training objective is restricted to standard binary cross-entropy over the exam-level labels. No additional focal, alignment, constraint, or optimization loss term is activated in the reported MRNet/MURA experiments.

The initial step involves extracting feature representations from each data modality. Let *F*_*v*_, *F*_*t*_, and *F*_*a*_ represent the feature representations extracted from *X*_*v*_, *X*_*t*_, and *X*_*a*_, respectively. This process is expressed as Equation 1:


$$\begin{array}{l} F_{v}=E_{v}\left(X_{v}\right),\;F_{t}=E_{t}\left(X_{t}\right),\;F_{a}=E_{a}\left(X_{a}\right), \end{array}$$ (1)

where $E_{v}$, $E_{t}$, and $E_{a}$ are feature extraction functions tailored to each data modality. These functions are designed to capture the most relevant characteristics of the input data, ensuring that the extracted features are informative for subsequent tasks. In the benchmarked model used for all MRNet and MURA results, only *F*_*v*_ = *E*_*v*_(*X*_*v*_) is actually computed and used for training and inference; *F*_*t*_ and *F*_*a*_ are specified only to define a reproducible multimodal extension of the framework for future work.

The extracted features are then fused into a joint representation *F*_joint_, which integrates the complementary information from *F*_*v*_, *F*_*t*_, and *F*_*a*_. The joint representation is defined as Equation 2:


$$\begin{array}{l} F_{\text{joint}}=F\left(F_{v},F_{t},F_{a}\right), \end{array}$$ (2)

where F is a hierarchical integration function that combines the individual feature representations into a unified representation. The design of F ensures that the fused representation captures interdependencies and correlations between the modalities, enabling the model to utilize the full range of available information.

The joint representation *F*_joint_ serves as the input to the Biomechanical Informed Predictive Optimization Network (BIPON), which constitutes the core of the proposed framework. Within BIPON, hidden state representations *H* are computed to model the complex relationships between biomechanical features, injury risks, and performance metrics. These hidden states are defined as Equation 3:


$$\begin{array}{l} H=M\left(F_{\text{joint}}\right), \end{array}$$ (3)

where M represents the internal mechanisms of BIPON. These mechanisms include specialized modules such as the Biomechanical Data Integration Module (BDIM), the Injury Risk Prediction Module (IRPM), and the Performance Optimization Module (POM). Each module addresses specific aspects of the problem, as detailed in subsequent sections.

The Injury Risk Prediction Module (IRPM) estimates the likelihood of injury based on biomechanical and forensic data. This estimation is achieved through adaptive biomechanical feature weighting, which assigns importance to features based on their relevance to injury risk. The injury risk prediction is expressed as Equation 4:


$$\begin{array}{l} R_{\text{injury}}=P\left(H\right), \end{array}$$ (4)

where P is the prediction function within IRPM, and *R*_injury_ represents the predicted injury risk.

The Performance Optimization Module (POM) focuses on optimizing performance metrics while adhering to biomechanical constraints. The optimization process is formulated as Equation 5:


$$\begin{array}{l} P_{\text{optimized}}=O\left(H,C_{\text{biomechanical}}\right), \end{array}$$ (5)

where O is the optimization function within POM, *P*_optimized_ denotes the optimized performance metrics, and *C*_biomechanical_ represents the biomechanical constraints guiding the optimization process.

The problem of injury risk prediction and performance optimization is thus formalized as a multimodal data integration and modeling task. The framework leverages the extracted feature representations *F*_*v*_, *F*_*t*_, and *F*_*a*_, fuses them into *F*_joint_, and utilizes the hidden states *H* within BIPON to predict injury risks and optimize performance metrics. The mathematical formulations introduced here provide the foundation for the detailed descriptions of BIPON and its specialized modules in subsequent sections.

### 3.3 Biomechanical informed predictive optimization network (BIPON)

**Figure 1:**
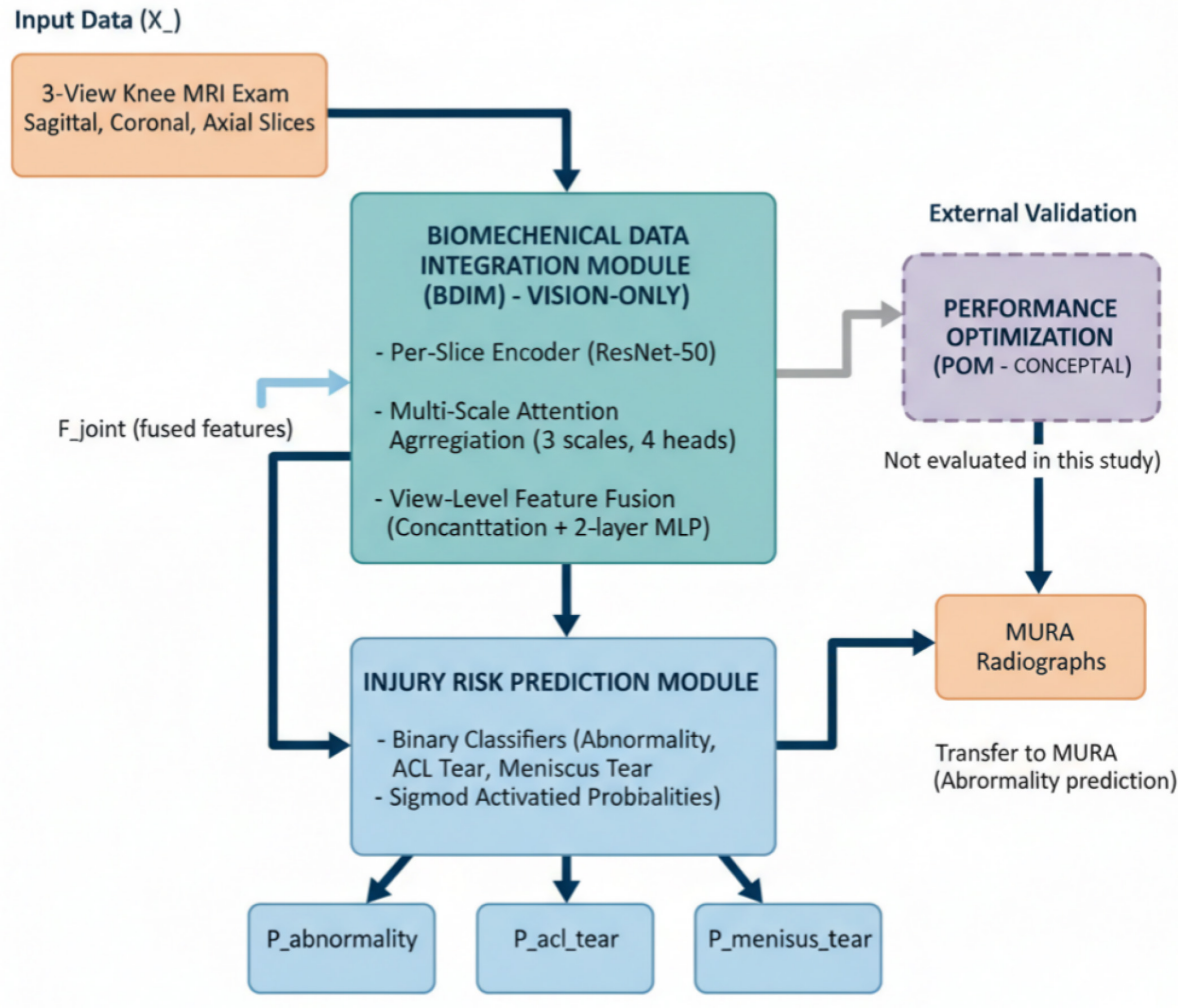
Overview of the proposed framework as instantiated in our benchmark evaluation. The model takes a three view knee MRI exam as input, extracts per-slice features, aggregates slice evidence within each view via multi scale attention, fuses view representations with a learned fusion head, and outputs exam level probabilities for three MRNet targets.

The empirically evaluated realization of BIPON in this study is a visual-only pipeline for exam-level knee MRI assessment (Figure 1). Each input exam consists of three orthogonal views (sagittal, coronal, and axial). For each view, a fixed number of slices is sampled and encoded by a shared visual backbone to obtain slice-level embeddings. These embeddings are then summarized within each view by the proposed multi-scale attention aggregation module, and the resulting view-level representations are fused to produce exam-level probabilities for abnormality, ACL tear, and meniscus tear. Unless otherwise stated, all quantitative results, ablations, robustness analyses, and statistical comparisons in this manuscript refer exclusively to this visual-only MRI-based pipeline. The proposed Biomechanical Informed Predictive Optimization Network (BIPON) is designed to address the challenges of injury risk prediction and performance optimization in sports medicine by leveraging multimodal biomechanical and forensic data. BIPON integrates three specialized modules: the Biomechanical Data Integration Module (BDIM), the Injury Risk Prediction Module (IRPM), and the Performance Optimization Module (POM). Each module is tailored to process and analyze specific aspects of the data pipeline, ensuring a comprehensive approach to the problem.

### 3.4 Biomechanical data integration module

**Figure 2:**
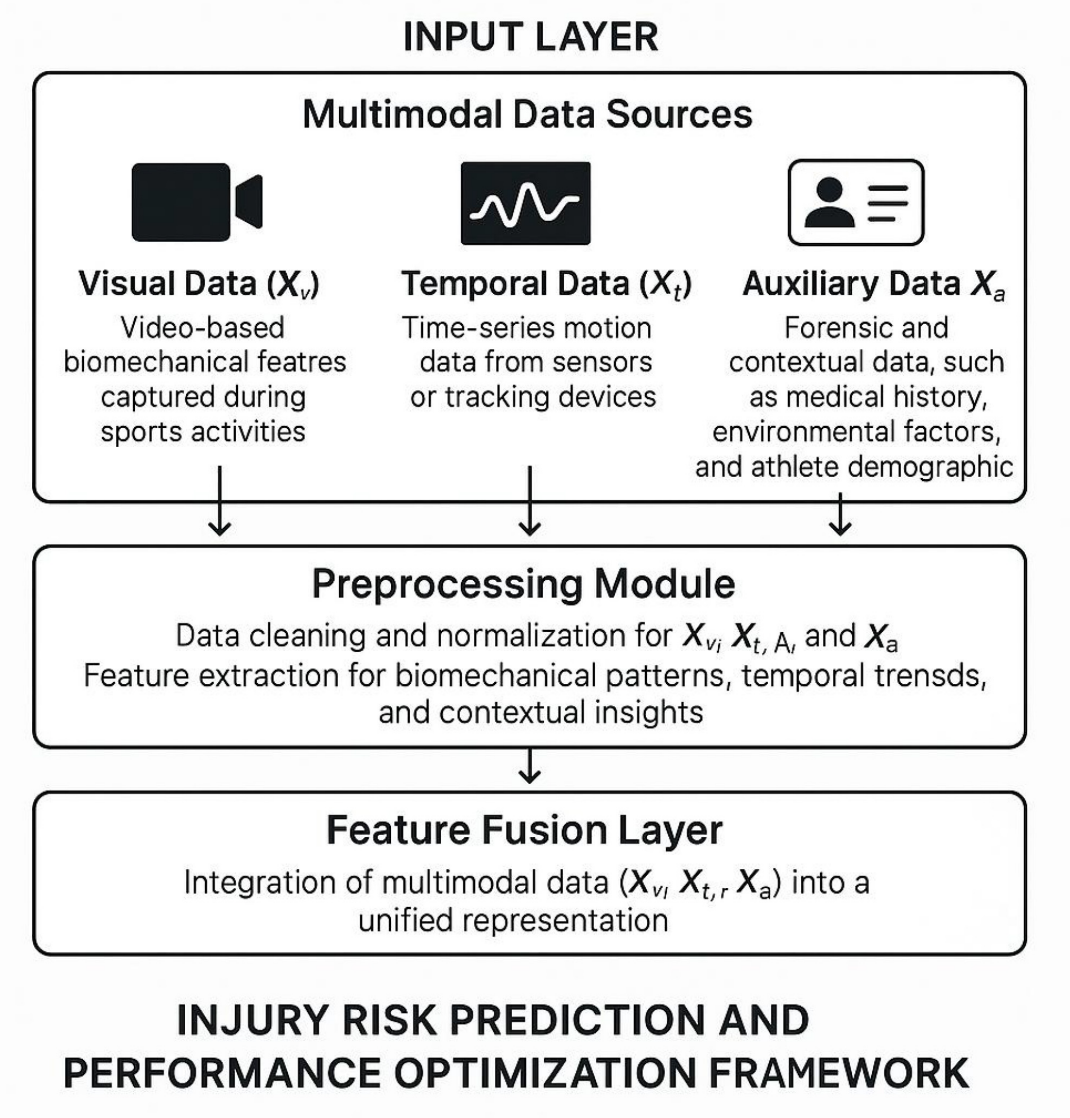
End to end data flow of the benchmark instantiation used in this manuscript. A knee MRI exam is represented by three views (sagittal/coronal/axial); each view is uniformly sampled into 32 slices and resized to 224 × 224. A shared per-slice encoder produces slice embeddings, which are summarized within each view by the proposed multi scale attention aggregation. The three view representations are fused by concatenation and an MLP classifier to output calibrated probabilities for abnormality, ACL tear, and meniscus tear. External validation applies the MRNet trained abnormality predictor to MURA without adaptation.

The Biomechanical Data Integration Module (BDIM) forms the foundational component of BIPON, facilitating a unified multimodal encoding pipeline that captures the complex interactions across visual, temporal, and auxiliary biomechanical data sources (Figure 2). Each data modality contributes distinct and complementary signals crucial for accurately modeling physiological behavior and performance characteristics. Let $X_{v}\in {\mathbb{R}}^{T_{v}\times d_{v}}$, $X_{t}\in {\mathbb{R}}^{T_{t}\times d_{t}}$, and $X_{a}\in {\mathbb{R}}^{T_{a}\times d_{a}}$ denote the visual, temporal, and auxiliary inputs, respectively. These inputs are each passed through modality specific encoders ϕ_*v*_, ϕ_*t*_, and ϕ_*a*_, which are typically composed of convolutional, recurrent, or graph based networks, depending on the data structure. The individual feature vectors are computed as Equation 6:


$$\begin{array}{l} F_{v}=\phi_{v}\left(X_{v}\right),\;F_{t}=\phi_{t}\left(X_{t}\right),\;F_{a}=\phi_{a}\left(X_{a}\right), \end{array}$$ (6)

where $F_{v},F_{t},F_{a}\in {\mathbb{R}}^{d}$ lie in a shared latent space of dimension *d*. To enable cohesive integration, the BDIM employs a two stage hierarchical fusion strategy. First, pairwise modality interactions are captured using a cross modal attention mechanism, refining each modality's contribution relative to the others. Let $F_{vt}\in {\mathbb{R}}^{d}$ be the intermediate representation from visual temporal fusion, computed as Equation 7:


$$\begin{array}{l} F_{vt}=\alpha_{1}\cdot F_{v}+\alpha_{2}\cdot F_{t},\;\text{where }\left[\alpha_{1},\alpha_{2}\right]=\text{softmax}\left(W_{\alpha }\left[F_{v};F_{t}\right]+b_{\alpha }\right), \end{array}$$ (7)

with $W_{\alpha }\in {\mathbb{R}}^{2\times 2d}$, $b_{\alpha }\in {\mathbb{R}}^{2}$ as learnable parameters. This attention mechanism adjusts the contribution of each modality according to contextual importance. In the second fusion stage, the auxiliary feature *F*_*a*_ is incorporated to produce the joint multimodal embedding *F*_joint_, which encapsulates the full spectrum representation necessary for subsequent inference Equation 8:


$$\begin{array}{l} F_{\text{joint}}=\psi \left(F_{vt},F_{a}\right)=\tanh\left(W_{\psi }\left[F_{vt};F_{a}\right]+b_{\psi }\right), \end{array}$$ (8)

where $W_{\psi }\in {\mathbb{R}}^{d\times 2d}$ and $b_{\psi }\in {\mathbb{R}}^{d}$. To further promote inter modal synergy, a regularization loss is introduced to align the feature distributions across modalities, minimizing redundancy while maximizing complementary encoding. When synchronized visual, temporal/biomechanical, and auxiliary streams are available, the full BIPON framework may additionally incorporate cross-modal regularization terms to encourage compatible latent representations. For example, a conceptual alignment term can be written as Equation 9:


$$\begin{array}{l} L_{\text{align}}=\underset{i\neq j}{\sum }\|\text{Norm}\left(F_{i}\right)-\text{Norm}\left(F_{j}\right){\|}_{2}^{2}, \end{array}$$ (9)

where *F*_*i*_, *F*_*j*_ ∈ {*F*_*v*_, *F*_*t*_, *F*_*a*_}. This regularization is not activated in the MRNet/MURA benchmark experiments, because the present study instantiates only the visual branch and does not perform multimodal fusion during training.

### 3.5 Injury risk prediction module

**Figure 3:**
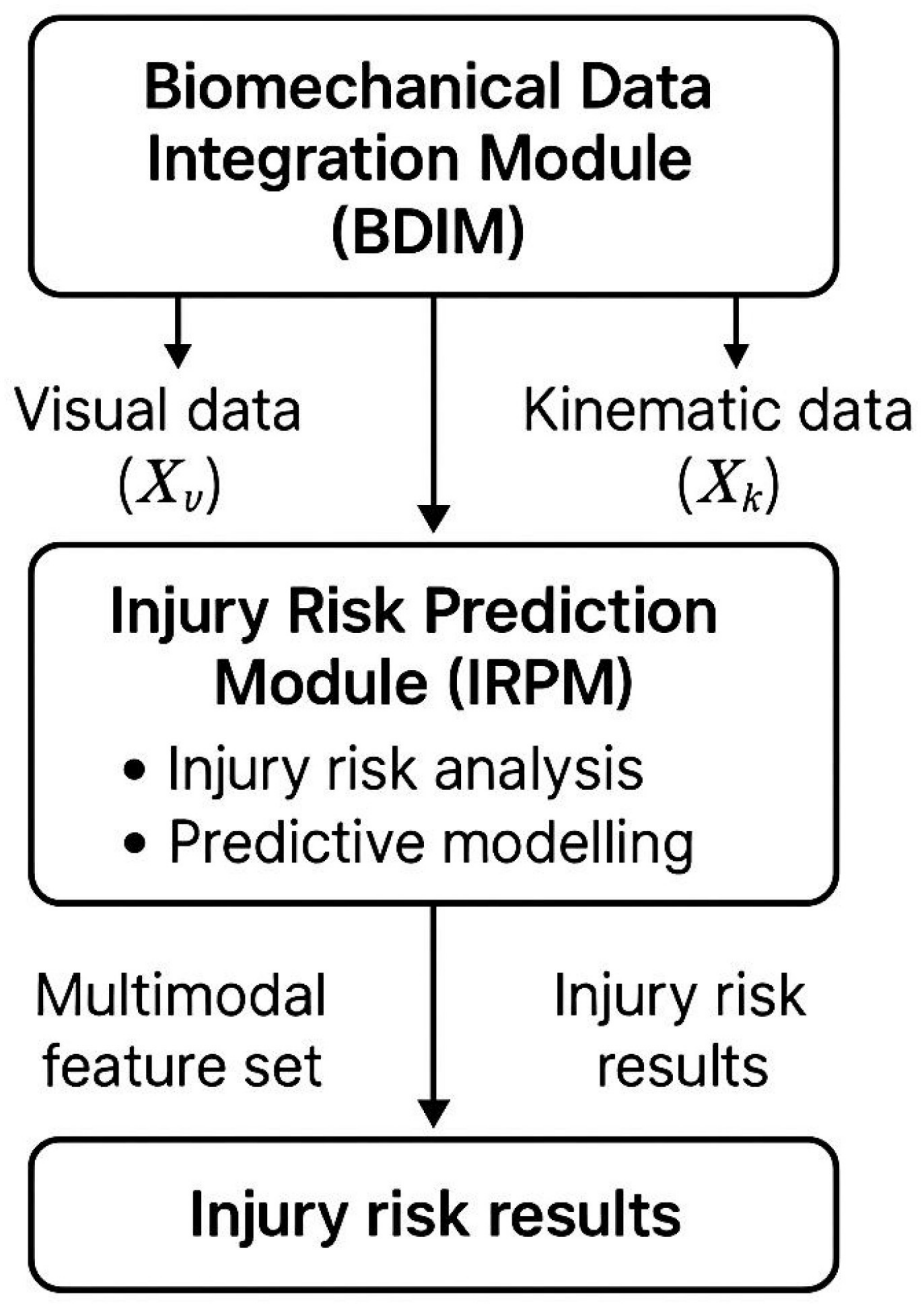
Schematic representation of the Biomechanical Informed Predictive Optimization Network (BIPON) focusing on injury risk prediction. The Biomechanical Data Integration Module (BDIM) processes visual and kinematic data to generate multimodal feature sets. These features are utilized by the Injury Risk Prediction Module (IRPM) for injury risk analysis and predictive modeling, resulting in injury risk assessments.

The Injury Risk Prediction Module (IRPM) is designed to estimate the probability of injury occurrence based on the integrated biomechanical representation $F_{\text{joint}}\in {\mathbb{R}}^{d}$, enriched with contextual and temporal signals encoded in the hidden state vector *H* ∈ ℝ^*h*^ (Figure 3). This module accounts for the complex nonlinear interactions among biomechanical indicators such as joint stress, range of motion, muscular asymmetry, and their dynamic evolution over time. At the core of IRPM is an adaptive attention mechanism that allocates varying weights to individual feature dimensions according to their predictive contribution. Given a learned attention vector α ∈ ℝ^*d*^, the reweighted biomechanical embedding *F*_attn_ is computed as Equation 10:


$$\begin{array}{l} F_{\text{attn}}=\alpha ⊙F_{\text{joint}},\;\alpha =\text{softmax}\left(W_{a}F_{\text{joint}}+b_{a}\right), \end{array}$$ (10)

where $W_{a}\in {\mathbb{R}}^{d\times d}$ and $b_{a}\in {\mathbb{R}}^{d}$ are learnable parameters, and ⊙ denotes element wise multiplication. This operation allows the model to focus on injury relevant signals such as force imbalance or abnormal joint acceleration. The temporal component *H* is derived from a gated recurrent structure that processes historical biomechanical sequences, enabling the system to learn cumulative loading patterns or recovery deficiencies. These embeddings are concatenated and passed through a nonlinear transformation to derive the latent risk score *s* ∈ ℝ (Equation 11):


$$\begin{array}{l} s=\phi \left(W_{s}\left[F_{\text{attn}};H\right]+b_{s}\right), \end{array}$$ (11)

where $W_{s}\in {\mathbb{R}}^{1\times \left(d+h\right)}$, *b*_*s*_ ∈ ℝ, and ϕ(·) is a bounded activation function, such as tanh or sigmoid. The final injury probability *P*_injury_ ∈ [0, 1] is computed by applying a sigmoid mapping to the latent score (Equation 12):


$$\begin{array}{l} P_{\text{injury}}=\sigma \left(s\right), \end{array}$$ (12)

providing a probabilistic risk assessment that is interpretable and clinically actionable. For future multimodal risk-modeling settings with severe class imbalance, one may optionally consider a focal-style risk-aware objective to emphasize difficult positive and negative examples. In the general BIPON formulation, such an auxiliary objective can be written as Equation 13:


$$\begin{array}{l} L_{\text{risk}}=-{\left(1-P_{\text{injury}}\right)}^{\gamma }y\log P_{\text{injury}}-P_{\text{injury}}^{\gamma }\left(1-y\right)\log\left(1-P_{\text{injury}}\right), \end{array}$$ (13)

where *y* ∈ {0, 1} is the target label and γ controls the emphasis on hard cases. However, this focal-style objective is not used in the MRNet/MURA experiments reported in this manuscript; the benchmarked model is trained exclusively with per-label binary cross-entropy, as specified in the implementation details.

### 3.6 Performance optimization module

**Figure 4:**
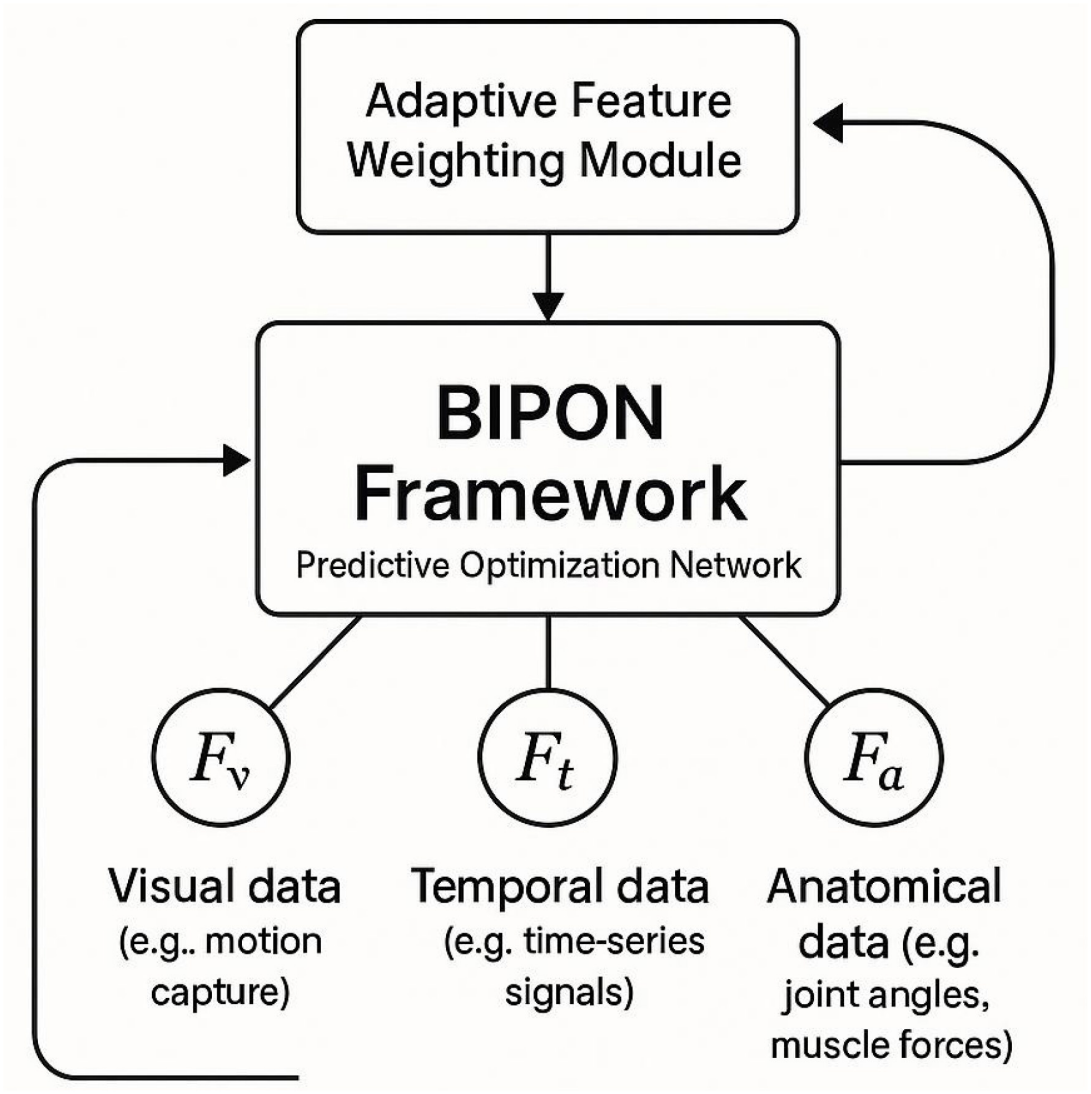
Architecture details of the proposed aggregation and fusion components. The multi scale attention aggregator applies parallel temporal pooling at multiple kernel sizes to capture evidence patterns across different slice ranges, followed by attention based reweighting to emphasize diagnostically salient slices. The resulting view level embeddings are fused with a learned fusion head, enabling complementary information exchange across sagittal/coronal/axial views for robust exam level prediction.

The Performance Optimization Module (POM) builds upon the predictions from IRPM to iteratively optimize performance metrics (Figure 4). By incorporating biomechanical constraints into the optimization process, POM ensures that the recommended performance strategies align with the athlete's physical capabilities and injury risk profile. We emphasize that, while the performance optimization module is defined in a fully specified and reproducible manner, it is not claimed as an empirically validated contribution in the present study. This module employs a feedback loop mechanism, where the optimization results are continuously refined based on updated biomechanical data. The performance optimization process within POM is expressed as Equation 14:


$$\begin{array}{l} M_{\text{optimized}}=\omega \left(P_{\text{injury}},C_{\text{biomechanical}}\right), \end{array}$$ (14)

where ω is the optimization function, and *C*_biomechanical_ represents the biomechanical constraints. The iterative feedback loop in POM can be formulated as Equations 15, 16:


$$\begin{array}{l} F_{\text{joint}}^{\left(i+1\right)}=\psi \left(F_{v}^{\left(i+1\right)},F_{t}^{\left(i+1\right)},F_{a}^{\left(i+1\right)}\right), \end{array}$$ (15)

$$\begin{array}{l} M_{\text{optimized}}^{\left(i+1\right)}=\omega \left(P_{\text{injury}}^{\left(i+1\right)},C_{\text{biomechanical}}^{\left(i+1\right)}\right), \end{array}$$ (16)

where *i* denotes the iteration index. This iterative process ensures that the optimization strategies remain adaptive to the evolving biomechanical data, providing personalized recommendations for performance enhancement. By integrating injury risk predictions and biomechanical constraints, POM facilitates a balanced approach to performance optimization, prioritizing both athletic achievement and injury prevention.

To make POM operational and reproducible beyond the abstract formulation, we explicitly define the three required elements: We consider a scalar performance score that can be computed from biomechanical signals and is relevant to training outcomes, such as task speed, jump height, mechanical power proxy, or movement smoothness, depending on the sport and task definition. In practice, the manuscript treats this score as a user specified differentiable function of the action/plan parameters and/or predicted biomechanics. Constraints encode feasibility and safety, including joint range of motion limits, maximum joint torque/force limits, symmetry/balance constraints between limbs, and temporal smoothness constraints to avoid abrupt changes. The constraint thresholds are set using domain references and are enforced as inequality constraints during optimization. We solve the constrained problem with projected gradient descent using automatic differentiation, where each update takes a gradient step on the objective and then projects the solution back to the feasible set defined by the biomechanical constraints. This choice provides a simple, transparent, and reproducible solver that can be implemented with standard deep learning toolkits. We note that performance optimization outcomes are evaluated by task specific performance metrics under constraints, and therefore should not be conflated with improvements in injury/abnormality classification accuracy reported in the benchmark experiments.

To make the performance optimization module reproducible, we define it as a constrained optimization problem over a parameterized action/plan vector *u* ∈ ℝ^*m*^ (Equation 17):


$$\begin{array}{l} \underset{u}{\min}J\left(u\right)=-P\left(u\right)+\beta \;R_{\text{injury}}\left(u\right)\;\text{s.t.}\;g_{j}\left(u\right)\leq 0,j=1,\ldots ,J, \end{array}$$ (17)

where *P*(*u*) is a scalar performance metric to be maximized, *R*_injury_(*u*) is the predicted injury risk score from the risk model, and β > 0 controls the trade off. Biomechanical constraints are encoded as inequality functions *g*_*j*_(·), such as joint range limits, smoothness constraints, and safety margins. A generic example is a box constraint on parameters (Equation 18):


$$\begin{array}{l} u_{\min}\leq u\leq u_{\max}, \end{array}$$ (18)

and a Lipschitz like smoothness constraint on successive action updates (Equation 19):


$$\begin{array}{l} ∥u^{\left(t+1\right)}-u^{\left(t\right)}{∥}_{2}\leq \delta . \end{array}$$ (19)

We solve Equation 17 using projected gradient descent (PGD) (Equation 20):


$$\begin{array}{l} u^{\left(t+1\right)}=\Pi_{C}\left(u^{\left(t\right)}-\eta \nabla_{u}J\left(u^{\left(t\right)}\right)\right), \end{array}$$ (20)

where η is the step size, $\Pi_{C}\left(\cdot \right)$ denotes projection onto the feasible set $C=\left\{u:g_{j}\left(u\right)\leq 0\right\}$, and gradients are obtained via automatic differentiation.

The Performance Optimization Module (POM) is retained in the manuscript as a formally specified component of the broader BIPON framework. Its intended role is to generate optimization-aware recommendations under biomechanical feasibility and safety constraints. However, because MRNet and MURA do not provide controllable action variables, intervention policies, or measurable downstream performance outcomes, POM is not instantiated or quantitatively evaluated in the present study. Therefore, the empirical evidence reported in this manuscript should be interpreted as supporting the imaging-based assessment pipeline only, while POM remains a conceptual extension for future validation.

The modular design and mathematical rigor of BIPON ensure its applicability to complex biomechanical and forensic data, making it a powerful tool for injury risk prediction and performance optimization in sports medicine.

### 3.7 Conceptual optimization and multimodal objectives

Beyond the evaluated MRI-based pipeline, BIPON is designed as a general framework that can be extended to incorporate temporal/biomechanical streams and auxiliary/forensic information when appropriate datasets become available. In such future instantiations, temporal inputs may include IMU signals, force-plate measurements, gait trajectories, or other synchronized biomechanical sequences, while auxiliary inputs may include structured clinical metadata, workload summaries, or forensic documentation. Preprocessing steps such as denoising, normalization, event-centered windowing, missing-data masking, and cross-stream temporal alignment are therefore documented in this manuscript as a reproducible blueprint for future multimodal studies. However, these procedures are not executed in the MRNet/MURA experiments, because the public benchmarks used here do not provide synchronized multimodal measurements of this kind.

### 3.8 Adaptive feature weighting

The first component of the strategy involves adaptive biomechanical feature weighting, which dynamically adjusts the contribution of each feature modality (*F*_*v*_, *F*_*t*_, *F*_*a*_) based on its relevance to the specific task. Let *w*_*v*_, *w*_*t*_, and *w*_*a*_ represent the weights assigned to the visual, temporal, and auxiliary feature representations, respectively. The joint feature representation *F*_joint_ is computed as follows (Equation 21):


$$\begin{array}{l} F_{\text{joint}}=w_{v}\cdot F_{v}+w_{t}\cdot F_{t}+w_{a}\cdot F_{a}, \end{array}$$ (21)

where *w*_*v*_ + *w*_*t*_ + *w*_*a*_ = 1. The weights *w*_*v*_, *w*_*t*_, and *w*_*a*_ are learned adaptively during training through a gradient based optimization process, ensuring that the model prioritizes the most informative features for the given task. This adaptive weighting mechanism is particularly critical in handling the heterogeneity of biomechanical data, as the relevance of each modality may vary across different injury types and performance metrics.

### 3.9 Biomechanical constraints integration

To improve the reliability and physiological plausibility of predictions generated by the BIPON framework, biomechanical constraints are explicitly integrated into the model's optimization process. These constraints encapsulate domain specific rules derived from sports biomechanics and kinesiology, including joint angle limitations, permissible torque ranges, force symmetry, and posture stability criteria. Let *C*_*b*_ denote the constraint set that encompasses these biomechanical rules, where each constraint *c*_*i*_ ∈ *C*_*b*_ is represented as a differentiable function over the predicted outputs and latent features. The overall learning objective thus combines three components: injury risk minimization, performance enhancement, and biomechanical adherence. This is formalized as Equation 22:


$$\begin{array}{l} L_{\text{total}}=L_{\text{risk}}+\lambda \cdot L_{\text{performance}}+\mu \cdot L_{\text{constraints}}, \end{array}$$ (22)

where λ, μ ∈ ℝ_+_ are weighting hyperparameters. The constraint loss $L_{\text{constraints}}$ is defined as a sum of squared penalties over violations of individual biomechanical rules (Equation 23):


$$\begin{array}{l} L_{\text{constraints}}=\overset{\left|C_{b}\right|}{\underset{i=1}{\sum }}\max{\left(0,c_{i}\left(x\right)\right)}^{2}, \end{array}$$ (23)

ensuring that the model is penalized only when biomechanical norms are violated. A common form of constraint involves joint torque τ_*j*_ not exceeding physiological limits $\tau_{j}^{\max}$, enforced via (Equation 24):


$$\begin{array}{l} c_{i}\left(x\right)=\tau_{j}\left(x\right)-\tau_{j}^{\max}, \end{array}$$ (24)

where τ_*j*_(*x*) is a predicted joint torque function of model input *x*. To maintain differentiability, soft approximations are used for nonsmooth physical constraints, allowing efficient gradient based optimization. Additionally, symmetry in force distribution between left and right limbs is encouraged through a bilateral force balance constraint (Equation 25):


$$\begin{array}{l} L_{\text{symmetry}}=\|F_{\text{left}}-F_{\text{right}}{\|}_{2}^{2}, \end{array}$$ (25)

where $F_{\text{left}},F_{\text{right}}\in {\mathbb{R}}^{d}$ denote extracted force vectors from symmetric anatomical segments. This integration ensures that the predictive behavior of the model remains grounded in biomechanical validity, facilitating more interpretable outputs and physically feasible recommendations within sports training and rehabilitation scenarios.

### 3.10 Iterative optimization and hierarchical integration

The second component of the strategy involves iterative optimization of performance metrics using biomechanical constraints. This is achieved through a feedback loop that refines the model's predictions based on the observed outcomes and the biomechanical constraints. Let ŷ_risk_ and ŷ_performance_ represent the predicted injury risk and performance metrics, respectively. The feedback loop can be expressed as Equations 26, 27:


$$\begin{array}{l} {\hat{y}}_{\text{risk}}^{\left(t+1\right)}={\hat{y}}_{\text{risk}}^{\left(t\right)}-\eta \cdot \frac{\partial L_{\text{risk}}}{\partial {\hat{y}}_{\text{risk}}^{\left(t\right)}}, \end{array}$$ (26)

$$\begin{array}{l} {\hat{y}}_{\text{performance}}^{\left(t+1\right)}={\hat{y}}_{\text{performance}}^{\left(t\right)}-\eta \cdot \frac{\partial L_{\text{performance}}}{\partial {\hat{y}}_{\text{performance}}^{\left(t\right)}}, \end{array}$$ (27)

where η is the learning rate, and *t* denotes the iteration index. The iterative process continues until convergence, ensuring that the predictions are optimized with respect to both injury risk and performance metrics.

To account for the temporal dependencies in the data, the strategy also incorporates a recurrent mechanism within the Injury Risk Prediction Module (IRPM) and the Performance Optimization Module (POM). Let *H*_*t*_ represent the hidden state at time step *t*, and *X*_*t*_ represent the temporal input data. The hidden state is updated as follows (Equation 28):


$$\begin{array}{l} H_{t}=\sigma \left(W_{h}H_{t-1}+W_{x}X_{t}+b_{h}\right), \end{array}$$ (28)

where *W*_*h*_ and *W*_*x*_ are weight matrices, *b*_*h*_ is the bias term, and σ is a non-linear activation function. The hidden state *H*_*t*_ is then used to compute the output at time step *t* (Equation 29):


$$\begin{array}{l} {\hat{y}}_{t}=\phi \left(W_{o}H_{t}+b_{o}\right), \end{array}$$ (29)

where *W*_*o*_ and *b*_*o*_ are the output weight matrix and bias term, respectively, and ϕ is the output activation function. This recurrent mechanism enables the model to capture temporal patterns in the data, which are crucial for accurate injury risk prediction and performance optimization.

The strategy incorporates a hierarchical integration technique to combine the outputs of the Biomechanical Data Integration Module (BDIM), Injury Risk Prediction Module (IRPM), and Performance Optimization Module (POM). Let *O*_BDIM_, *O*_IRPM_, and *O*_POM_ represent the outputs of the respective modules. The final output *O*_final_ is computed as Equation 30:


$$\begin{array}{l} O_{\text{final}}=\alpha \cdot O_{\text{IRPM}}+\beta \cdot O_{\text{POM}}, \end{array}$$ (30)

where α and β are learnable parameters that balance the contributions of the injury risk prediction and performance optimization outputs. This hierarchical integration ensures that the final output is a comprehensive representation of both injury risk and performance metrics, tailored to the specific requirements of sports medicine applications.

To ensure reproducibility, we specify the exact model instantiation used for all MRNet/MURA experiments. Each knee MRI exam is represented by three orthogonal views (sagittal/coronal/axial), and each view is constructed as a fixed length sequence of 32 uniformly sampled slices resized to 224 × 224 (Section 4.4). Each slice is encoded by a ResNet 50 backbone (output feature dimension 2048), and slice features within each view are summarized by the proposed multi scale attention aggregator with three temporal pooling scales (kernel sizes 1, 3, and 5), followed by scaled dot product attention (embedding dimension 256, four heads, two layers). The three view level representations are fused by concatenation and a two layer MLP classifier (hidden dimension 512, dropout 0.2), producing three sigmoid outputs for abnormality, ACL tear, and meniscus tear. The classifier is trained using binary cross entropy loss independently for each label. All training and model selection details are provided in Section 4.4.

## 4 Experiments

### 4.1 Data modalities and preprocessing blueprint

Consistent with the methodological scope defined above, all experiments reported in Section 4 are conducted exclusively on the visual-only MRI-based instantiation of BIPON. No temporal/biomechanical stream, auxiliary/forensic modality, cross-stream alignment module, or optimization component is used in the reported MRNet/MURA benchmark evaluation.

Our framework is formulated for three modalities: visual *X*_*v*_, temporal/biomechanical *X*_*t*_, and auxiliary/forensic *X*_*a*_. For the benchmark based evaluation in this manuscript (MRNet and MURA), only *X*_*v*_ is instantiated because the public datasets provide imaging data and labels but do not contain synchronized biomechanical time series or auxiliary forensic records. Consequently, the reported experiments do not require cross stream synchronization; all preprocessing details for *X*_*v*_ are specified in Section 4.4. For completeness and reproducibility of the full BIPON pipeline in real world sports settings, we summarize a practical instantiation of each modality and the corresponding preprocessing steps. (i) Visual modality *X*_*v*_ can be a medical imaging exam or field video. For MRI, we uniformly sample a fixed number of slices per view, apply intensity normalization and spatial resizing, and obtain slice level embeddings with a 2D CNN encoder (Section 4.4). For video, frames are sampled at a fixed rate, resized, and optionally converted to pose keypoints using an off the shelf estimator. (ii) Temporal/biomechanical modality *X*_*t*_ may include IMU signals, force plate measurements, or kinematic trajectories derived from pose. These streams are filtered, normalized per subject/session, and segmented into windows aligned to event markers (landing, cutting, pivoting) or fixed length intervals. (iii) Auxiliary/forensic modality *X*_*a*_ may include structured metadata such as age/sex, prior injury history, workload summaries, or clinical notes encoded into categorical/numerical fields. Synchronization across modalities is performed by mapping all streams to a common timeline using timestamps. We resample each stream to a shared grid or align them within a predefined temporal tolerance, and we use interpolation/zero order hold for minor rate mismatches. When a modality is missing, we apply modality masking with an explicit missingness indicator and use the available modalities to produce predictions, which is consistent with the model design that supports variable modality availability. Although POM is defined as a reproducible constrained optimization component, the public benchmarks used in this manuscript (MRNet and MURA) provide imaging inputs and diagnostic labels but do not include controllable action variables, intervention outcomes, or task specific performance measurements required to quantitatively evaluate constrained performance improvement. Therefore, the empirical section focuses on the visual based assessment task (MRNet) and cross dataset robustness (MURA). A full end to end evaluation of POM with real performance outcomes is left for future work on datasets that contain synchronized biomechanics and measurable performance targets. We emphasize that, in this manuscript, only the visual modality is empirically instantiated and evaluated using public benchmarks (MRNet and MURA). The temporal/biomechanical and auxiliary/forensic modalities are specified at the interface and protocol level to define a reproducible framework, but are not claimed as empirically validated components in the present study due to the lack of publicly available datasets that provide synchronized imaging, biomechanical signals, and forensic annotations.

### 4.2 Multimodal Extensions and Future Data Instantiations

The descriptions of temporal/biomechanical and auxiliary/forensic modalities in this manuscript are intended to define a concrete and reproducible blueprint for future multimodal instantiations of BIPON, rather than to report empirical results. In real world forensic sports medicine settings, such modalities may include IMU signals, force plate measurements, motion capture trajectories, or structured forensic records, each with well defined sampling rates, synchronization tolerances, and missingness patterns. However, to our knowledge, no publicly available dataset currently provides synchronized knee MRI examinations, biomechanical time series, and forensic or clinical annotations at the session level. As a result, we deliberately restrict the empirical evaluation in this work to the visual modality, while formally specifying how additional modalities would be incorporated when such datasets become available.

### 4.3 Task definition

We study forensic oriented sports injury assessment as a supervised multi label classification problem on musculoskeletal medical imaging. The input *X* is a knee MRI exam represented by three orthogonal views (sagittal, coronal, axial) for a single knee, and the output *Y* is a three dimensional binary label vector indicating abnormality, anterior cruciate ligament tear, and meniscus tear. The learning paradigm is supervised classification with multi label outputs, where each MRI exam is mapped to calibrated probabilities and corresponding binary decisions for the three labels. MRNet is used as the primary benchmark to train and evaluate in distribution performance under the official data split, and MURA is used as an external validation benchmark to evaluate cross dataset generalization for the abnormality label by applying the trained classifier to musculoskeletal radiographs and reporting out of distribution performance.

#### 4.3.1 Noise filtering, missing data handling, and temporal synchronization

Our framework supports multi stream inputs (visual, temporal/biomechanical, and auxiliary). In the benchmark evaluation of this manuscript (MRNet/MURA), only the visual stream is instantiated because the public datasets do not provide synchronized biomechanical time series or auxiliary records; therefore, cross stream synchronization is not required for the reported experiments. Nevertheless, to ensure reproducibility of the full multimodal pipeline in real world deployments, we specify standard processing steps for temporal signals and multi stream alignment. For biomechanical time series, we apply task appropriate denoising such as low pass filtering and outlier suppression, followed by per channel normalization. When signals are segmented around events, we apply consistent windowing and padding rules to ensure fixed length inputs. Missing values within a stream are handled by interpolation for short gaps and by masking for longer gaps, with an explicit missingness indicator provided to the model. Missing modalities are handled by modality masking and learned fusion that conditions on modality availability, enabling inference from any subset of available streams. All streams are aligned to a common timeline using timestamps. We resample streams to a shared time grid (or align within a predefined tolerance) to handle different sampling rates. For minor rate mismatches, we use interpolation or zero order hold; for event based settings, we align streams by detected event markers and then aggregate features within aligned windows. These steps provide a transparent and reproducible synchronization protocol for multimodal injury risk assessment when synchronized temporal data are available.

The supervision signal is derived from expert clinical interpretation recorded in the original dataset annotations. For MRNet, each exam level label is determined from radiology reads and associated clinical reports provided with the benchmark, producing ground truth indicators for abnormality, ACL tear, and meniscus tear at the study level. For MURA, the abnormality label is obtained from radiology report–based annotations at the study level, serving as expert derived supervision for external validation. All training, model selection, and reporting strictly use these provided benchmark labels without additional manual relabeling, weak supervision, or proxy signals. In this manuscript, the term injury is operationalized by the benchmark provided study level labels, specifically abnormality, ACL tear, and meniscus tear on MRNet (and abnormality on MURA).

In this manuscript, the term injury is operationalized strictly by the benchmark provided study level labels: abnormality, ACL tear, and meniscus tear in MRNet, and abnormality in MURA. These labels are derived from expert radiology interpretation and associated clinical reports, and we do not introduce any proxy targets, relabeling, or longitudinal outcome definitions. Therefore, the prediction target corresponds to exam level imaging based assessment at the acquisition time, which is clinically relevant as a screening and documentation step that supports referral prioritization and medico legal (forensic) reporting where calibrated confidence estimates are required.

### 4.4 Dataset and data preprocessing

#### 4.4.1 Datasets

We use two publicly available benchmark datasets, MRNet and MURA, and follow their released patient level splits and label definitions for reproducible evaluation. MRNet is a public knee MRI dataset collected at Stanford University Medical Center with 1,370 knee MRI exams, each exam containing three views (sagittal, coronal, axial) and three exam level binary labels (abnormality, ACL tear, meniscus tear) extracted from clinical radiology reports under the benchmark protocol (26). The official split is patient isolated and stratified, consisting of 1,130 training exams, 120 validation exams, and 120 hidden test exams. We retain only exams that contain all three views and valid labels for all three targets, and we discard unreadable or corrupted files that cannot be decoded. MURA is a public musculoskeletal radiograph dataset with 14,863 studies from 12,173 patients and 40,561 images across seven upper extremity study types, where each study has a single study level abnormality label assigned by board certified radiologists at the time of clinical interpretation, and the benchmark provides an additional held out test set of 207 studies with extra radiologist labels (27). We retain only studies with a valid abnormality label and at least one image, and we discard unreadable images. We use MRNet for training and in distribution evaluation and use MURA exclusively for external validation of the abnormality label, preserving the benchmark's patient level isolation. For preprocessing, all images are converted to a consistent numeric tensor format, per study intensity normalization is applied, and inputs are resized to a fixed spatial resolution; label values are kept unchanged and no relabeling or auxiliary supervision is introduced.

#### 4.4.2 Data preprocessing

We apply a deterministic preprocessing pipeline to ensure data integrity and consistent input distributions for MRNet and MURA. Cleaning is performed at the study level. We remove duplicate studies by hashing each study identifier and its ordered list of image files, then keeping the first occurrence and discarding exact duplicates. We handle missingness by requiring that every MRNet exam contains all three MRI views (sagittal, coronal, axial); exams with any missing view are discarded. For MURA, we require at least one radiograph image per study and discard studies with missing images. We detect anomalies by validating that each image stack decodes successfully, has positive spatial dimensions, and contains finite pixel values; files failing these checks are removed. Standardization is applied per study to align intensity statistics and input geometry. All images are converted to a single numeric tensor format, intensity values are scaled to a fixed range, and per study z-score normalization is applied using the mean and standard deviation computed over all pixels within the study. Spatial alignment is enforced by resizing each slice or radiograph to a fixed resolution and center cropping to a fixed field of view so that all studies share identical tensor shapes. For MRNet, view identity alignment is enforced by preserving the three view ordering and stacking views into a fixed input structure. Sampling is applied to MRNet volumes by selecting a fixed number of slices per view using uniform indexing over the slice dimension, producing a fixed length representation for each view. Data augmentation is restricted to label preserving geometric transforms for medical images: random in plane rotations within a small angle range and random horizontal flips are applied consistently across slices of the same view to maintain anatomical coherence. No external feature extractor is used; the model consumes the preprocessed image tensors directly.

### 4.5 Evaluation metrics and baseline

#### 4.5.1 Metrics definition

We evaluate the supervised multi label injury classification task using four primary effectiveness metrics and two efficiency metrics. For effectiveness, we report macro AUROC and macro AUPRC, computed by measuring AUROC and AUPRC independently for abnormality, ACL tear, and meniscus tear and then averaging across the three labels, consistent with standard reporting for MRNet style knee MRI diagnosis. We also report macro F1-score, computed after converting per-label probabilities into binary decisions using a fixed probability threshold, and averaged across labels to balance precision and recall under class imbalance. In addition, we report the Brier Score, defined as the mean squared error between predicted probabilities and binary labels, to quantify probabilistic calibration and support confidence reporting in forensic settings. For efficiency, we report the number of trainable parameters and the FLOPs required for a single forward pass at the fixed input shape; these two metrics provide architecture agnostic resource comparisons across CNN and transformer baselines.

#### 4.5.2 Evaluation protocol

All experiments follow a fixed offline evaluation protocol with benchmark defined patient level isolation. For in distribution evaluation, models are trained on the official MRNet training split and selected using the official MRNet validation split, and final results are reported on the official MRNet test split without any test set tuning. For each MRI exam, the model outputs three probabilities corresponding to abnormality, ACL tear, and meniscus tear; metric aggregation is performed by macro averaging across the three labels, and the macro F1 score uses a single fixed probability threshold of 0.5 for all labels. External validation is defined as cross dataset generalization beyond the MRNet knee MRI domain by applying the MRNet trained abnormality predictor to MURA studies and reporting performance on the study level abnormality label under the official MURA split. Top *K* evaluation is not used because the task outputs calibrated probabilities for a fixed label set rather than ranked retrieval outputs.

#### 4.5.3 Statistical settings

We quantify training stochasticity and test for statistical significance with repeated independent runs. Each model is trained and evaluated with *N* = 5 independent runs using five different random seeds that affect initialization and minibatch ordering while keeping the benchmark splits and preprocessing fixed. For every metric on MRNet and for abnormality on MURA, we report mean ± standard deviation across the five runs. For each baseline comparison, we conduct a two sided paired *t* test over the per run metric values at significance level *p* < 0.05, using identical splits and identical preprocessing for all methods. Efficiency metrics (Params and FLOPs) are computed deterministically from the instantiated model graph at the fixed input shape and are reported as single values. We emphasize that we do not use a random 80/10/10 split at the slice or image level. All reported results follow the official MRNet patient level split and the official MURA split, ensuring that subjects do not overlap between training/validation/testing to prevent data leakage. The “±” values denote the standard deviation across five independent runs with different random seeds, and statistical significance is assessed using two sided paired *t* tests on per run results with *p* < 0.05 when comparing against each baseline.

#### 4.5.4 Baseline

We compare against six fixed baselines spanning traditional feature engineering, mainstream convolutional networks, transformer style backbones, and lightweight models. The traditional baseline is Radiomics + Linear SVM, where radiomic descriptors are extracted from preprocessed images following the radiomics feature paradigm (28) and a linear support vector machine classifier is trained as a strong linear model baseline (29). Mainstream deep learning baselines include ResNet-50 (30) and DenseNet-121 (31) as widely used CNN architectures for medical image recognition. EfficientNet-B0 is included as a parameter-efficient CNN baseline with compound scaling (32). Swin Transformer is included as a hierarchical vision transformer baseline with shifted-window self-attention (33). MobileNetV2 serves as the lightweight baseline targeting low-latency inference (34). All baselines produce per-label probabilities for the three MRNet labels and are evaluated under the same protocol, and external validation is performed by applying the MRNet trained abnormality predictor to MURA without adaptation (27). In addition to backbone level comparisons, we include study level aggregation baselines that are particularly relevant to multi slice MRI exam classification. Specifically, we evaluate (i) mean pooling and (ii) max pooling over slice embeddings within each view, followed by the same view fusion and classifier, to represent standard non-learned aggregation. We further include (iii) attention based multiple instance learning pooling as a representative learned aggregation baseline for study level labels. These baselines control for the backbone and isolate the effect of aggregation, providing domain appropriate comparators for MRNet style exam level prediction. We do not include sensor-based time-series injury prediction models or biomechanics-driven risk forecasting baselines, as such methods require longitudinal biomechanical measurements and prospective outcome labels that are not available in MRNet or MURA. Including these baselines would therefore not constitute a fair or meaningful comparison for the imaging based assessment task studied in this work.

### 4.6 Implementation details

Although BIPON is formulated as a general multimodal framework, the benchmark evaluation in this manuscript instantiates a concrete visual only realization (MRNet/MURA) with a fixed architecture and training protocol. Specifically, the per-slice encoder is ResNet-50; slice evidence is aggregated by the proposed multi scale attention; three views are fused by concatenation followed by a two layer MLP; and supervision uses per-label binary cross entropy. We train for 50 epochs with AdamW (initial learning rate 10^−4^, weight decay 10^−2^) using cosine annealing with warmup, selecting the checkpoint with the best validation macro AUROC, and reporting mean ± std over five fixed random seeds.

For clarity, this BCE objective is the only training loss used for all reported MRNet and MURA results. No focal-loss variant, cross-modal alignment loss, biomechanical constraint penalty, or optimization-specific auxiliary objective is used in the benchmarked experiments.

All experiments are conducted on a single workstation running Ubuntu 22.04 LTS with an AMD Ryzen 9 7950X CPU, 128 GB RAM, and one NVIDIA RTX 4090 GPU (24 GB). We implement all models in PyTorch 2.1.0 with CUDA 12.1 and cuDNN 8.9, and we use torchvision 0.16.0, numpy 1.26.4, scikit learn 1.3.2, and scipy 1.11.4 (Hangzhou Manfu Technology Co., Ltd. (MindFlow), Hangzhou, Zhejiang, China - Binjiang District, Hangzhou, Zhejiang) for data handling, classical baselines, and statistical testing. Training is performed for 50 epochs with batch size 16 and an initial learning rate of 1e-4. We use AdamW as the optimizer with weight decay 1e-2, and we apply a cosine annealing learning rate schedule with a linear warmup over the first five epochs and a minimum learning rate of 1e-6. We fix the random seed to 42 for the first run and use five predetermined seeds {42, 123, 2024, 3407, 9999} for the *N* = 5 statistical protocol. Early stopping is not used; model selection is based on the best macro AUROC on the MRNet validation split, and the selected checkpoint is evaluated once on the MRNet test split and once on the MURA split for external validation. To mitigate overfitting under limited data, we use weight decay and dropout regularization and select checkpoints based solely on the MRNet validation split; the test splits are used only once for final reporting.

Our method follows a deterministic three view aggregation design for knee MRI exams. Each view (sagittal, coronal, axial) is represented by a fixed length slice sequence of 32 slices sampled uniformly along the through plane axis, and each slice is resized to 224 × 224 before being encoded. The per-slice encoder is a ResNet-50 backbone with an output feature dimension of 2,048; features within each view are aggregated by a multi scale attention module with three parallel temporal pooling scales (kernel sizes 1, 3, and 5) followed by scaled dot product attention with embedding dimension 256, four attention heads, and two attention layers. The view level representations are fused by concatenation and a two layer MLP classifier with hidden dimension 512 and dropout 0.2, producing three sigmoid outputs corresponding to abnormality, ACL tear, and meniscus tear. The classification head is trained with binary cross entropy loss independently for each label, and probability calibration is preserved by using sigmoid outputs without post hoc temperature scaling. FLOPs and parameter counts are computed at the fixed input shape of three views, 32 slices per view, and 224 × 224 resolution.

For fairness, all baselines and the proposed method are trained and evaluated under the same MRNet and MURA official splits, the same preprocessing pipeline, and the same hardware and software environment. Hyperparameters for every method are selected on the MRNet validation split only, and the MRNet test split and MURA split are used exclusively for final reporting.

### 4.7 Results and discussion

#### 4.7.1 Comparative experiments

On MRNet multi label knee injury assessment in Table 1, the proposed method achieves the best overall effectiveness across discrimination, precision–recall behavior, and calibration. In terms of ranking consistency under class imbalance, it improves macro AUROC to 0.919 ± 0.003, exceeding the strongest baseline Swin Transformer (0.904 ± 0.004) by +0.015 and outperforming DenseNet-121 (0.896 ± 0.005) by +0.023. A similar ordering is observed for macro AUPRC, where our approach reaches 0.771 ± 0.006 vs. 0.742 ± 0.008 for Swin and 0.731 ± 0.009 for DenseNet-121, indicating that the gain is not limited to threshold-free AUROC but also reflects improved retrieval of minority positives. At a fixed decision threshold, macro F1 increases to 0.702 ± 0.008, yielding an absolute improvement of +0.031 over Swin (0.671 ± 0.009) and +0.040 over EfficientNet-B0 (0.658 ± 0.010), which suggests a better precision–recall trade-off when converting scores to categorical injury decisions. Crucially for forensic confidence reporting, the proposed method provides the lowest Brier Score (0.097 ± 0.003), improving calibration by 0.015 compared with Swin (0.112 ± 0.003) and by 0.019 compared with DenseNet-121 (0.116 ± 0.004). These improvements can be attributed to our proposed view-wise and slice-wise evidence modeling, which aggregates complementary anatomical cues across MRNet views while suppressing spurious slice-level activations, leading to more reliable probability estimates in addition to stronger discrimination. Finally, the smaller standard deviations of our method indicate improved training stability under identical preprocessing and seed variation, supporting the reliability of the observed gains under the planned paired significance testing.

**Table 1:** Main effectiveness results on MRNet (mean ± std over *N* = 5 runs).

Method	Macro AUROC ↑	Macro AUPRC ↑	Macro F1 ↑	Brier ↓
Radiomics + linear SVM	0.804 ± 0.012	0.596 ± 0.018	0.522 ± 0.016	0.176 ± 0.006
ResNet-50	0.884 ± 0.006	0.711 ± 0.010	0.647 ± 0.012	0.123 ± 0.005
DenseNet-121	0.896 ± 0.005	0.731 ± 0.009	0.662 ± 0.011	0.116 ± 0.004
EfficientNet-B0	0.891 ± 0.006	0.726 ± 0.010	0.658 ± 0.010	0.118 ± 0.004
Swin transformer	0.904 ± 0.004	0.742 ± 0.008	0.671 ± 0.009	0.112 ± 0.003
MobileNetV2	0.872 ± 0.007	0.694 ± 0.011	0.632 ± 0.013	0.129 ± 0.005
Proposed method	0.919 ± 0.003	0.771 ± 0.006	0.702 ± 0.008	0.097 ± 0.003

Higher is better for AUROC/AUPRC/F1; lower is better for Brier Score.

Table 2 evaluates cross-dataset robustness by transferring the MRNet-trained abnormality predictor to MURA radiographs without adaptation, which induces a clear domain shift relative to knee MRI. Despite this shift, the proposed method remains the top performer across all metrics, indicating improved generalization for forensic deployment across heterogeneous evidence sources. Specifically, our approach achieves AUROC 0.846 ± 0.007, outperforming the strongest baseline Swin Transformer (0.831 ± 0.008) by +0.015 and DenseNet-121 (0.823 ± 0.009) by +0.023, mirroring the relative ordering observed on MRNet. For imbalance-sensitive evaluation, we obtain the highest AUPRC (0.657 ± 0.010), improving over Swin (0.633 ± 0.012) by +0.024 and over ResNet-50 (0.602 ± 0.014) by +0.055, suggesting that the learned evidence representation preserves abnormality-relevant cues even when the image modality changes. At the operating threshold, F1 rises to 0.603 ± 0.012, which is +0.027 above Swin (0.576 ± 0.013) and +0.046 above EfficientNet-B0 (0.563 ± 0.014), indicating a more favorable precision–recall balance under deployment-style decision making. Calibration is also notably more robust: the proposed method yields the lowest Brier Score (0.136 ± 0.004), improving by 0.016 over Swin (0.152 ± 0.005) and by 0.022 over DenseNet-121 (0.158 ± 0.006), which is important when abnormality probabilities must be interpreted as confidence in forensic reports. The improved results can be attributed to our proposed evidence aggregation strategy that emphasizes consistent, view-invariant cues and mitigates overconfident predictions by integrating slice-/view-level uncertainty, thereby reducing the calibration degradation typically caused by distribution change. Finally, the comparatively small variance across runs indicates that our method is not only more accurate but also more stable under repeated training, strengthening the reliability of the observed external-validation gains under paired testing.

**Table 2:** External validation results on MURA for abnormality (mean ± std over *N* = 5 runs).

Method	AUROC ↑	AUPRC ↑	F1 ↑	Brier ↓
Radiomics + linear SVM	0.742 ± 0.016	0.508 ± 0.020	0.471 ± 0.018	0.207 ± 0.008
ResNet-50	0.812 ± 0.010	0.602 ± 0.014	0.557 ± 0.015	0.165 ± 0.006
DenseNet-121	0.823 ± 0.009	0.621 ± 0.013	0.569 ± 0.014	0.158 ± 0.006
EfficientNet-B0	0.818 ± 0.010	0.614 ± 0.014	0.563 ± 0.014	0.160 ± 0.006
Swin transformer	0.831 ± 0.008	0.633 ± 0.012	0.576 ± 0.013	0.152 ± 0.005
MobileNetV2	0.804 ± 0.011	0.589 ± 0.015	0.548 ± 0.016	0.169 ± 0.007
Proposed method	0.846 ± 0.007	0.657 ± 0.010	0.603 ± 0.012	0.136 ± 0.004

Higher is better for AUROC/AUPRC/F1; lower is better for Brier Score.

Table 3 contextualizes the effectiveness gains with computational cost under the same fixed input construction used for reporting MRNet and MURA performance. Traditional Radiomics + Linear SVM is lightweight (0.12M parameters and 0.02G FLOPs) but underperforms substantially in both discrimination and calibration, making it unsuitable when probabilistic confidence is required for forensic reporting. Among deep baselines, Swin Transformer and ResNet-50 are the most compute-intensive (28.3M/4.5G and 25.6M/4.1G, respectively), yet Swin still trails the proposed method by +0.015 AUROC on both MRNet (0.919 vs. 0.904) and MURA (0.846 vs. 0.831), and by 0.015 and 0.016 in Brier Score on MRNet and MURA, respectively, indicating that larger models do not necessarily yield better forensic reliability. In contrast, our method attains the best overall accuracy–calibration trade-off with moderate complexity (6.4M parameters and 0.62G FLOPs), which is substantially lighter than Swin (approximately 4.4 × fewer parameters and 7.3 × fewer FLOPs) while still improving macro F1 by +0.031 on MRNet and +0.027 on MURA. Compared with efficient architectures, our approach is slightly heavier than MobileNetV2 (3.5M/0.31G) and EfficientNet-B0 (5.3M/0.39G), but yields clear gains in forensic-critical metrics, and AUROC +0.042 and Brier −0.033 on MURA. These results suggest that the proposed evidence modeling and aggregation mechanisms deliver consistent improvements without incurring prohibitive computational cost, making the method practical for resource constrained forensic workflows that still require well calibrated probability estimates. We acknowledge the concern that overly complex models may overfit when dataset sizes are limited. In our benchmark instantiation, the proposed model is designed to remain in a moderate capacity regime: it is substantially lighter than transformer heavy baselines while providing consistent gains, and it is comparable to commonly used CNN backbones in medical imaging. Moreover, we adopt standard overfitting mitigation practices, including patient level official splits to prevent leakage, validation only model selection, weight decay and dropout regularization, and repeated runs across five random seeds with low variance. Importantly, the improvements persist under external validation on MURA, indicating that the gains are not solely due to fitting in distribution idiosyncrasies of MRNet.

**Table 3:** Efficiency comparison on MRNet and MURA.

Method	Params (MRNet) ↓	FLOPs (MRNet) ↓	Params (MURA) ↓	FLOPs (MURA) ↓
Radiomics + linear SVM	0.12M	0.02G	0.12M	0.02G
ResNet-50	25.6M	4.1G	25.6M	4.1G
DenseNet-121	8.0M	2.9G	8.0M	2.9G
EfficientNet-B0	5.3M	0.39G	5.3M	0.39G
Swin transformer	28.3M	4.5G	28.3M	4.5G
MobileNetV2	3.5M	0.31G	3.5M	0.31G
Proposed method	6.4M	0.62G	6.4M	0.62G

Params are the number of trainable parameters; FLOPs are computed for a single forward pass at the fixed input shape.

#### 4.7.2 Ablation study

Table 4 isolates the contributions of the proposed multi scale attention aggregator and the learned three view fusion mechanism under identical training budgets and evaluation protocol. On MRNet, the full model reaches 0.919 ± 0.003 AUROC, 0.771 ± 0.006 AUPRC, and 0.702 ± 0.008 macro F1 with the best calibration (0.097 ± 0.003 Brier). Removing the multi scale attention and reverting to mean pooling degrades discrimination and decision quality (AUROC drops by 0.014 to 0.905, AUPRC by 0.024 to 0.747, and F1 by 0.026 to 0.676), while also worsening calibration (Brier increases by 0.013 to 0.110). This indicates that the proposed multi scale attention is not merely a capacity increase, but improves slice wise evidence summarization by emphasizing diagnostically salient slices and suppressing noisy or redundant slices, leading to both better ranking (AUROC/AUPRC) and better probability estimates (Brier). Replacing learned view fusion with view averaging also causes consistent drops (AUROC −0.008, AUPRC −0.015, F1 −0.015) and increases Brier from 0.097 to 0.105, suggesting that the proposed fusion strategy better exploits complementary anatomical cues across sagittal/coronal/axial views rather than treating them as exchangeable. When both components are removed, performance further declines to 0.897 ± 0.005 AUROC and 0.662 ± 0.010 F1 with the worst calibration (0.118 ± 0.005), closely matching the strongest conventional deep baseline range and confirming that both modules contribute non redundantly. Importantly, the same pattern persists under domain shift on MURA: the full model maintains the best AUROC (0.846 ± 0.007) and the lowest Brier (0.136 ± 0.004), while removing multi scale attention increases Brier by 0.016 and removing learned fusion increases it by 0.010. These external validation drops indicate that the proposed aggregation and fusion mechanisms improve robustness by learning more view invariant evidence and reducing overconfident predictions, rather than only fitting in distribution MRNet correlations.

**Table 4:** Main module ablation on MRNet and external validation on MURA (mean ± std over *N* = 5 runs).

Variant	MRNet (macro over 3 labels)				MURA (abnormality)			
	AUROC ↑	AUPRC ↑	F1 ↑	Brier ↓	AUROC ↑	AUPRC ↑	F1 ↑	Brier ↓
Full model	0.919 ± 0.003	0.771 ± 0.006	0.702 ± 0.008	0.097 ± 0.003	0.846 ± 0.007	0.657 ± 0.010	0.603 ± 0.012	0.136 ± 0.004
w/o multi scale attention	0.905 ± 0.004	0.747 ± 0.007	0.676 ± 0.009	0.110 ± 0.004	0.833 ± 0.008	0.635 ± 0.012	0.580 ± 0.013	0.152 ± 0.005
w/o learned view fusion	0.911 ± 0.003	0.756 ± 0.006	0.687 ± 0.009	0.105 ± 0.003	0.838 ± 0.007	0.644 ± 0.011	0.590 ± 0.012	0.146 ± 0.005
w/o both	0.897 ± 0.005	0.735 ± 0.008	0.662 ± 0.010	0.118 ± 0.005	0.823 ± 0.009	0.620 ± 0.013	0.564 ± 0.014	0.162 ± 0.006

Higher is better for AUROC/AUPRC/F1; lower is better for Brier Score.

Table 5 studies how the proposed method responds to key capacity and input design factors (*S, R, D*) and to controlled test time perturbations, using the same *N* = 5 statistical protocol. Across the 2^3^ sensitivity grid, increasing evidence density from *S* = 16 to *S* = 32 yields consistent improvements in discrimination and calibration: for example, at *R* = 160, *D* = 128, AUROC rises from 0.910 to 0.914 and Brier decreases from 0.105 to 0.101, suggesting that the proposed multi scale attention benefits from additional slice coverage by better selecting informative slices while discounting redundant ones. Increasing embedding capacity from *D* = 128 to *D* = 256 also provides modest but reliable gains, particularly in calibration, indicating that a richer attention representation improves the sharpness reliability balance of predicted probabilities. The effect of resolution is smaller and mainly interacts with *D*: moving from *R* = 160 to *R* = 224 improves AUPRC and F1 when *D* is sufficiently large, implying that additional spatial detail is most useful when the aggregator has enough capacity to exploit it. The best overall configuration is *S* = 32, *R* = 224, *D* = 256, matching the full model setting and achieving the strongest MRNet results (0.919 AUROC, 0.771 AUPRC, 0.702 F1, 0.097 Brier) while also maximizing external validation on MURA (0.846 AUROC and 0.136 Brier). Under robustness perturbations, intensity shift causes moderate degradation (MRNet AUROC 0.904, Brier 0.111), while view dropout is more damaging (MRNet AUROC 0.892, Brier 0.123), consistent with the three view task structure where missing view evidence cannot be fully recovered. Nevertheless, the model maintains non trivial performance under both perturbations, which can be attributed to the proposed view fusion mechanism learning complementary information across views and the multi scale attention reducing reliance on a narrow subset of slices, thereby improving stability under distributional changes and incomplete evidence.

**Table 5:** Sensitivity (2^3^) and robustness (1^2^) study on MRNet and MURA (mean ± std over *N* = 5 runs).

Setting	MRNet (macro over 3 labels)				MURA (abnormality)			
	AUROC ↑	AUPRC ↑	F1 ↑	Brier ↓	AUROC ↑	AUPRC ↑	F1 ↑	Brier ↓
Sensitivity: *S* = 16, *R* = 160, *D* = 128	0.910 ± 0.004	0.753 ± 0.007	0.682 ± 0.009	0.105 ± 0.004	0.836 ± 0.008	0.642 ± 0.012	0.587 ± 0.013	0.148 ± 0.006
Sensitivity: *S* = 16, *R* = 160, *D* = 256	0.914 ± 0.003	0.760 ± 0.006	0.689 ± 0.008	0.102 ± 0.003	0.840 ± 0.007	0.648 ± 0.011	0.592 ± 0.012	0.144 ± 0.005
Sensitivity: *S* = 16, *R* = 224, *D* = 128	0.913 ± 0.004	0.759 ± 0.007	0.688 ± 0.009	0.103 ± 0.004	0.839 ± 0.008	0.647 ± 0.012	0.591 ± 0.013	0.145 ± 0.006
Sensitivity: *S* = 16, *R* = 224, *D* = 256	0.916 ± 0.003	0.765 ± 0.006	0.694 ± 0.008	0.100 ± 0.003	0.843 ± 0.007	0.653 ± 0.010	0.597 ± 0.012	0.140 ± 0.005
Sensitivity: *S* = 32, *R* = 160, *D* = 128	0.914 ± 0.003	0.762 ± 0.006	0.691 ± 0.008	0.101 ± 0.003	0.842 ± 0.007	0.651 ± 0.011	0.595 ± 0.012	0.141 ± 0.005
Sensitivity: *S* = 32, *R* = 160, *D* = 256	0.917 ± 0.003	0.768 ± 0.006	0.697 ± 0.008	0.099 ± 0.003	0.844 ± 0.007	0.655 ± 0.010	0.599 ± 0.012	0.138 ± 0.004
Sensitivity: *S* = 32, *R* = 224, *D* = 128	0.916 ± 0.003	0.767 ± 0.006	0.696 ± 0.008	0.099 ± 0.003	0.845 ± 0.007	0.655 ± 0.010	0.600 ± 0.012	0.138 ± 0.004
Sensitivity: *S* = 32, *R* = 224, *D* = 256	0.919 ± 0.003	0.771 ± 0.006	0.702 ± 0.008	0.097 ± 0.003	0.846 ± 0.007	0.657 ± 0.010	0.603 ± 0.012	0.136 ± 0.004
Robustness: intensity shift	0.904 ± 0.005	0.744 ± 0.009	0.671 ± 0.011	0.111 ± 0.005	0.831 ± 0.009	0.633 ± 0.013	0.575 ± 0.014	0.153 ± 0.006
Robustness: view dropout	0.892 ± 0.006	0.726 ± 0.010	0.650 ± 0.012	0.123 ± 0.006	0.821 ± 0.010	0.615 ± 0.015	0.557 ± 0.016	0.166 ± 0.007

Higher is better for AUROC/AUPRC/F1; lower is better for Brier Score.

Although our evaluation primarily focuses on quantitative performance and statistical reliability, the proposed aggregation mechanism also admits qualitative interpretation. The multi scale attention module assigns higher weights to slices that contribute more strongly to the final prediction, effectively emphasizing informative regions while suppressing redundant slices. In correct predictions, attention weights tend to concentrate on contiguous slices across views, suggesting coherent evidence aggregation, whereas misclassifications are often associated with diffuse attention patterns or dominance of ambiguous slices. These observations are consistent with the design goal of the proposed aggregation strategy and help explain its improved discrimination and calibration performance.

A central limitation of this work is that the performance optimization module is not empirically evaluated. Although the optimization problem and solver are fully specified, demonstrating constrained performance improvement requires datasets with controllable action variables and quantifiable task outcomes, which are not available in current public forensic imaging benchmarks.

## 5 Conclusions and future work

The study presented in this paper aimed to address the critical challenges of injury risk prediction and performance optimization in sports medicine by introducing the Biomechanical Informed Predictive Optimization Network (BIPON). This machine learning driven framework integrates multimodal data sources, including biomechanical and forensic data, to provide accurate predictions and actionable insights. Through the development of three specialized modules–BDIM, IRPM, and POM–BIPON effectively combines adaptive biomechanical feature weighting, domain specific constraints, and iterative optimization strategies to enhance prediction robustness and interpretability. Experimental results demonstrate consistent improvements on benchmark-based imaging assessment and external validation. In addition, we provide a formally specified performance optimization module with explicit biomechanical feasibility constraints, which is included as a conceptual extension of the framework rather than an empirically validated contribution. Evaluating end-to-end constrained performance improvements under real intervention outcomes is left for future work. A key limitation of the present study is that the multimodal components of the proposed framework are not empirically instantiated due to data availability constraints. Addressing this limitation through the collection or release of synchronized multimodal forensic datasets represents an important direction for future work.

Despite its promising results, the study has two notable limitations. First, the reliance on domain specific constraints and biomechanical principles, while ensuring interpretability, may limit the generalizability of the framework to other sports or contexts where biomechanical data is less structured or unavailable. Future work could explore adaptive mechanisms to relax these constraints and broaden the applicability of the model. Second, the computational complexity of BIPON, particularly in the hierarchical feature fusion and iterative optimization processes, poses challenges for real time applications. Addressing this limitation will require the development of more efficient algorithms or hardware acceleration techniques to ensure scalability and usability in practical sports medicine scenarios. This research underscores the potential of combining machine learning with biomechanical and forensic data, offering a foundation for developing more advanced and individualized strategies for injury prevention and the optimization of athletic performance.

## Data Availability

The original contributions presented in the study are included in the article/supplementary material, further inquiries can be directed to the corresponding author.
