# Correction: Forensic-oriented injury and abnormality assessment in sports medicine via a biomechanically-informed predictive framework

**DOI:** 10.3389/fmed.2026.1912615

**Published:** 2026-07-02

**Authors:** Xiaolin Wang, Liduan Zheng, Zeyu Li

**Affiliations:** 1 Department of Pathology, Union Hospital, Tongji Medical College, Huazhong University of Science and Technology, Wuhan, China; 2 School of Life Sciences, Northeast Forestry University, Harbin, China

**Keywords:** biomechanical optimization, injury risk prediction, machine learning, multimodal data integration, sports medicine

Author Zeyu Li was erroneously assigned as corresponding author. The correct corresponding author is Liduan Zheng.

The original version of this article has been updated.

